# Localized and Long-Lasting Adaptation in Dragonfly Target-Detecting Neurons

**DOI:** 10.1523/ENEURO.0036-24.2024

**Published:** 2024-09-17

**Authors:** Matthew B. Schwarz, David C. O'Carroll, Bernard J. E. Evans, Joseph M. Fabian, Steven D. Wiederman

**Affiliations:** ^1^School of Biomedicine, The University of Adelaide, Adelaide, South Australia 5001, Australia; ^2^Department of Biology, Lund University, Lund 22100, Sweden

**Keywords:** adaptation, dragonfly, small target motion detection neurons

## Abstract

Some visual neurons in the dragonfly (*Hemicordulia tau*) optic lobe respond to small, moving targets, likely underlying their fast pursuit of prey and conspecifics. In response to repetitive targets presented at short intervals, the spiking activity of these “small target motion detector” (STMD) neurons diminishes over time. Previous experiments limited this adaptation by including intertrial rest periods of varying durations. However, the characteristics of this effect have never been quantified. Here, using extracellular recording techniques lasting for several hours, we quantified both the spatial and temporal properties of STMD adaptation. We found that the time course of adaptation was variable across STMD units. In any one STMD, a repeated series led to more rapid adaptation, a minor accumulative effect more akin to habituation. Following an adapting stimulus, responses recovered quickly, though the rate of recovery decreased nonlinearly over time. We found that the region of adaptation is highly localized, with targets displaced by ∼2.5° eliciting a naive response. Higher frequencies of target stimulation converged to lower levels of sustained response activity. We determined that adaptation itself is a target-tuned property, not elicited by moving bars or luminance flicker. As STMD adaptation is a localized phenomenon, dependent on recent history, it is likely to play an important role in closed-loop behavior where a target is foveated in a localized region for extended periods of the pursuit duration**.**

## Significance Statement

The dragonfly is an effective and efficient predator, with specialized target-detecting neurons located within the brain's optic lobe. When presented with repeated targets, the spiking activity of these target-detecting neurons is reduced. Such adaptation to repeated stimulation is a common property of neurons across diverse species. Our results show that target-induced adaptation is constrained to the location of the presented targets. Furthermore, we have quantified the degree to which neuronal responses to moving targets are reduced and then recover over time. This adaptation in a visual feature-discrimination pathway raises important questions about the functional implications of neuronal adaptation on the crucial behavior of target pursuit.

## Introduction

Neuronal responses across diverse species change when exposed to prolonged or repeated stimuli, occurring over timescales from milliseconds (e.g., photoreceptors; Weckström, 1989) to a year (e.g., long-term potentiation; Abraham et al., 2002). This adaptation can fundamentally change both temporal and spatial characteristics of neural responses over time (Kohn, 2007; Weber et al., 2019; Benda, 2021). The term adaptation describes observable phenomena without implying specific mechanisms (Kohn, 2007; Benda, 2021), which could range from changes in transmembrane currents (Benda, 2021), modulation of synaptic input (Kohn, 2007; Nikolaev et al., 2013; Benda, 2021), to migration of screening pigment (Daw and Pearlman, 1974).

In response to luminance intensity, photoreceptor responses exhibit neuronal adaptation, encoding changes in intensity rather than ambient light levels (Weckström, 1989). This adjustment in gain alleviates saturation and enhances information transmission (Laughlin, 1989), suited to environments where contrast boundaries represent features, and overall light changes can be slight or in orders of magnitude (Van Hateren, 1997). Further downstream, mouse retinal ganglion cells adapt to luminance and contrast over multiple timescales, influenced by history. Here, the changing statistical properties of the stimulus over time infers the degree of neuronal adaptation, resulting in efficient encoding within the neurons’ output range (Wark et al., 2009).

In the insect, wide-field motion-detecting neurons adapt by reducing responses to constant movement, thereby extending velocity encoding within the neuron's dynamic range (Maddess and Laughlin, 1985; Harris et al., 1999; Fairhall et al., 2001; Barnett et al., 2010; D. C. O’Carroll et al., 2012; Evans et al., 2019). In the dragonfly's target-detection pathway, adaptation can both increase and decrease neuronal activity. In response to a continuously moving target, spike rates are facilitated, increasing over time (Nordström et al., 2011). While under strong stimulation, some small target motion detectors (STMDs) hyperpolarize reducing spiking activity, resulting in a postexcitatory inhibition of spontaneous activity (Fabian et al., 2019; Lancer et al., 2019; Fabian et al., 2023). Downstream from the optic lobe, neurons within the dragonfly ventral nerve cord respond to repeating targets with diminished activity that is unaltered by sensitizing stimuli (Olberg, 1981). Conversely, similar diminishing responses in the locust's descending contralateral movement detector can be modulated by external factors (Fraser Rowell, 1971).

Adaptation may be dependent on the spatial localization of the visual stimulus. For example, some insect visual neurons that exhibit reduced responses to repeating small moving targets show a naive response when the location of the target stimulus is relocated within the receptive field (Fraser Rowell, 1971; Olberg, 1981). While in larval tiger salamanders, retinal ganglion cells exhibit a more advanced form of spatial localization with antagonistic plasticity, characterized by central adaptation and peripheral sensitization (Kastner and Baccus, 2013).

Dragonflies are highly successful predators, detecting, tracking, and chasing prey with a high capture rate (Olberg et al., 2000; Combes et al., 2012). Likely to underlie these behaviors are the STMD neurons in the optic lobe (D. O’Carroll, 1993). STMDs are sensitive to target contrast, in addition to being tuned to the size and velocity of moving targets, even when embedded in cluttered environments (D. O’Carroll, 1993; Geurten et al., 2007; Wiederman et al., 2013; D. C. O’Carroll and Wiederman, 2014; Evans et al., 2022). They also display attention-like mechanisms, selecting and responding to a single target among pairs (Wiederman and O’Carroll, 2013; Lancer et al., 2019, 2022).

Here we characterize the spatiotemporal properties of adaptation in dragonfly STMD neurons by repeatedly presenting moving targets with intertrial intervals ranging from 1 s to 10 min. The results describe neuronal adaptation in the target-detecting visual pathway, enhancing our understanding of how dynamically varying neuronal responses underlie crucial behaviors like pursuit.

## Materials and Methods

### Experimental design

We used extracellular recordings from STMDs in 71 wild caught *Hemicordulia tau* (male and female). With the legs removed, the upright body was waxed to an articulating stand. The dragonfly head was waxed in position, tilted forward ∼60° to allow dissection and access to the posterior surface of the brain. A thin silver reference wire was inserted into the contralateral posterior side of the head capsule.

Extracellular probes (tungsten wire stereotrodes, 1 MΩ, World Precision Instruments, part # TST33C10KTH) were inserted into the ipsilateral lobula complex using an electronic micromanipulator (Sensapex UMTSC) mounted on a manual micromanipulator (WPI M3301R). Ringer’s solution (140 NaCl, 5 KCl, 5 MgCl_2_, 5 CaCl_2_, 3 NaHCO_3_, and 6.3 HEPES, pH 7.0; Fotowat et al., 2009) was perfused onto the brain surface via a right-lateral posterior incision to prevent desiccation. Delivery was through a microsyringe connected to polyimide tubing, with the tube end placed in the contralateral hole. Ringer was administered as needed during rest periods, perfusing the entire brain.

Electrodes were connected to an analog head stage amplifier (Neuralynx HS-18), and data were digitized at 32 kHz using a Neuralynx Digital Lynx SX (Cheetah) and a bespoke MATLAB data acquisition interface.

### Visual stimuli

Dragonflies were placed 20 cm from the stimulus monitor, centered on the visual midline (Asus VG279QM: 27”; IPS; 1,920 × 1,080 px; 280 Hz). The dorsal part of the head (fixed forward) was aligned with the top of the monitor. The screen extended 104° in azimuth and 58° in elevation (21 to 80° elevation from the eye's equator). OpenGL was used for projection distortion to ensure targets had constant angular dimensions and velocities. Stimuli were presented using a custom software package in MATLAB (RRID: SCR_001622) using Psychtoolbox (RRID: SCR_002881). An optical trigger (photodiode) synchronized data acquisition with stimulus generation.

Pictograms are illustrative and not to scale. Unless otherwise stated, adaptive visual stimuli consisted of small black targets (1.5° by 1.5°; 0.1 cd/m^2^) on a white background (382 cd/m^2^), moving rightward at 50°/s for 25° (i.e., within the neuron's excitatory receptive field).

### Identification of STMDs

We presented a series of visual stimuli to classify extracellular units. This characterizing series included moving texel patterns, drifting gratings, elongated bars, edges, drifting targets, and full-screen flicker. STMDs were identified by their robust response to small drifting targets and lack of response to larger moving features. We determined the neuron's receptive field by drifting small targets at varying locations across the monitor, in all four cardinal directions. STMD units reported herein all showed similar response characteristics to target velocity, size, contrast, and direction.

### Spike sorting

We filtered extracellular recordings with a second-order Butterworth bandpass filter (300–4,000 Hz). A detection threshold was manually set to isolate units using Plexon Offline Sorter with waveform length of 1,000 μs, a prethreshold period of 250 μs, and a deadtime of 1,000 μs. Spike sorting was conducted with either Valley Seeking Scan with a Parzen multiplier (0.5–2; step by 0.1 sorted in 2D space) or T-Dist-Em (degrees of freedom multiplier set at 10, 8 initial units).

### Data exclusion criterion

Sorted waveforms were visually inspected for unit clustering and allocation, utilizing metrics, refractory violations, clusters versus time view, cross-correlograms, and interspike interval histograms. We sorted and presented spiking activity from the dominant STMD unit. An exclusion criterion was applied to data if the characterizing stimuli indicated pathology or unit crossover. A pathological loss of a recording was indicated by naive responses (following a 10 min rest) below 60 spikes/s and inconsistent or high spontaneous activity. This preceded total cessation of neuronal responses and inconsistent responses to characterization stimuli.

### Data analysis and statistics

Spike sorted data were further analyzed using bespoke MATLAB scripts, tailored for each experiment. Unless otherwise noted, analysis windows for data reported were 250 ms in duration at the center of the target's trajectory. Spontaneous activity was quantified by counting spikes during the period 500 ms prior to each individual trial. Each dragonfly was considered an independent sample, noted within the figure legends as “*N*”, with “*n*” indicating technical replicates. We report effect size using Cohen's d (corrected with Hedges’ *d*, Jamovi software package). CI refers to the 95% confidence interval of the sample mean. Changes in response rate were curve fit in MATLAB (initial value, τ and plateau). Linear regression was calculated in GraphPad Prism 10.

## Results

**Figure 1.**
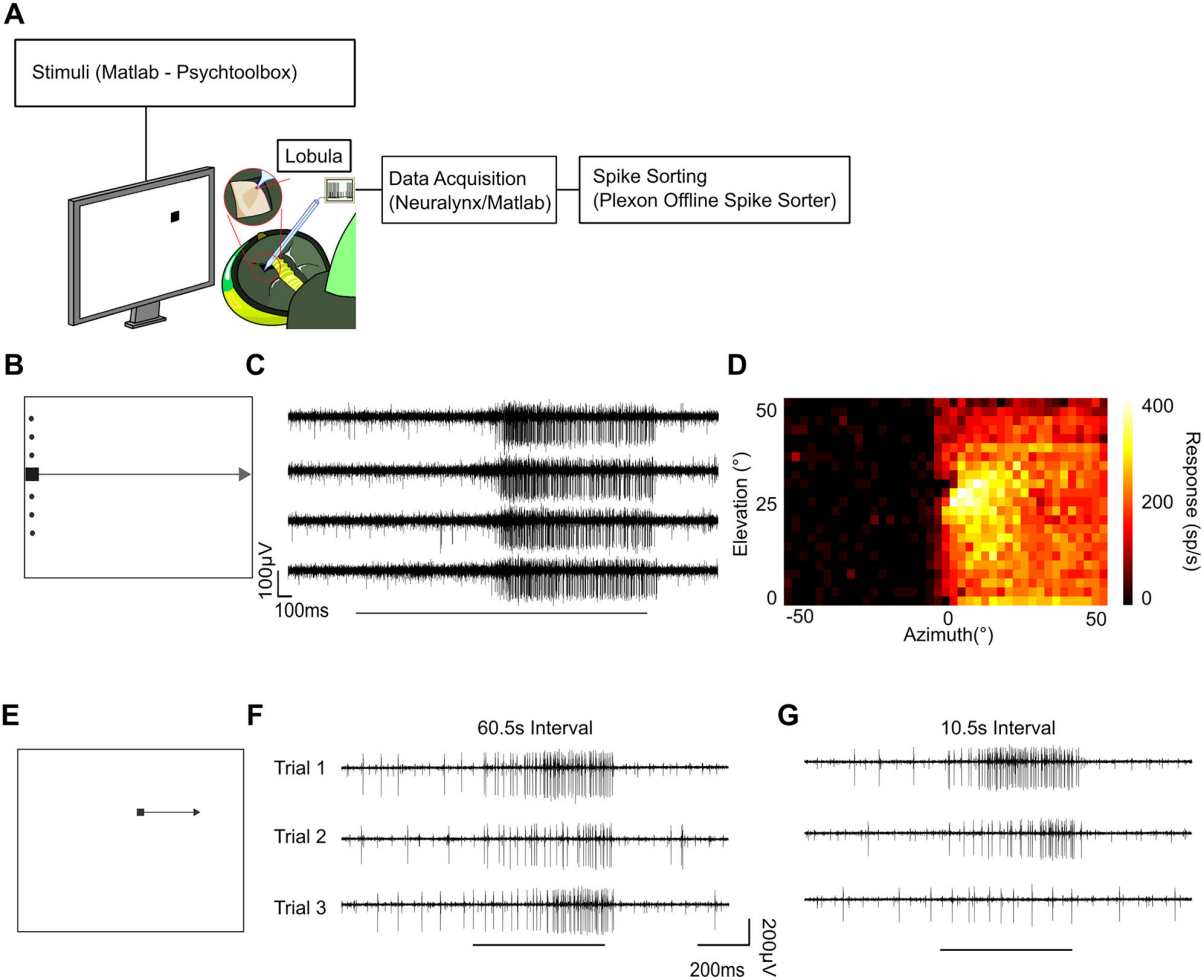
Dragonfly STMD neurons exhibit strong adaptation to a short-interval repetitive stimulus. ***A***, In vivo, extracellular techniques permitted long-lasting recordings of STMDs. ***B***, Pictogram illustrates mapping of the receptive field by presenting a series of small, dark targets drifting rightward (23 elevations vertically separated by 1.09°). ***C***, Four example traces at central locations of the STMD receptive field (black bar when target was on screen). ***D***, Colormap generated from the binned responses reveals STMD activity corresponding to target location across the visual field. ***E***, Pictogram illustrates the adapting stimulus consisting of a repeating drifting small dark target within the excitatory receptive field. ***F***, Example responses of three consecutive trials at 60.5 s intervals to targets presented at the adapting location (50°/s for 25°). ***G***, Three consecutive trials at 10.5 s intervals reveal stronger neuronal adaptation.

To quantify the spatiotemporal characteristics of adaptation, we performed in vivo extracellular recordings of STMD unit activity (Fig. 1*A*). We first mapped each STMD's receptive field by sequentially drifting small dark targets on a white background in each of the four cardinal directions (41 vertical and 23 horizontal trajectories at varying locations, where the order was pseudorandomized. Rightward is illustrated in Fig. 1*B*). Figure 1*C* shows exemplar data traces of responses to dark targets traversing four different rightward paths (at varying vertical offsets). To derive a receptive field, spiking activity was binned (100 ms) and plotted for each of the 23 paths (Fig. 1*D*). This receptive field reveals a robust excitatory response to targets presented in the contralateral hemifield and minimal response (almost spontaneous) to targets when moved within the ipsilateral hemifield (0° is the visual midline).

Based on the measured receptive field, we identified an “adapting” stimulus location (Fig. 1*E*) for repeated presentation of a single target along a 25° trajectory within the “hot spot” of the excitatory receptive field shown in Figure 1*D*. When presented at large (60.5 s) intervals, responses reveal typical neuronal variability (Fabian et al., 2023) while maintaining overall robust response (Fig. 1*F*). As expected from our prior work on facilitation in these neurons (Nordström et al., 2011), spiking responses to the 25° trajectory are initially weaker (unfacilitated) compared with those traversing the entire screen (Fig. 1*C*). Figure 1*G* shows individual examples of responses to targets presented at the same location at shorter (10.5 s) intervals. In this example, the repeated stimulus resulted in strong adaptation to near spontaneous levels, in as few as three trials (Fig. 1*G*).

### STMD responses to rapidly repeated targets

**Figure 2.**
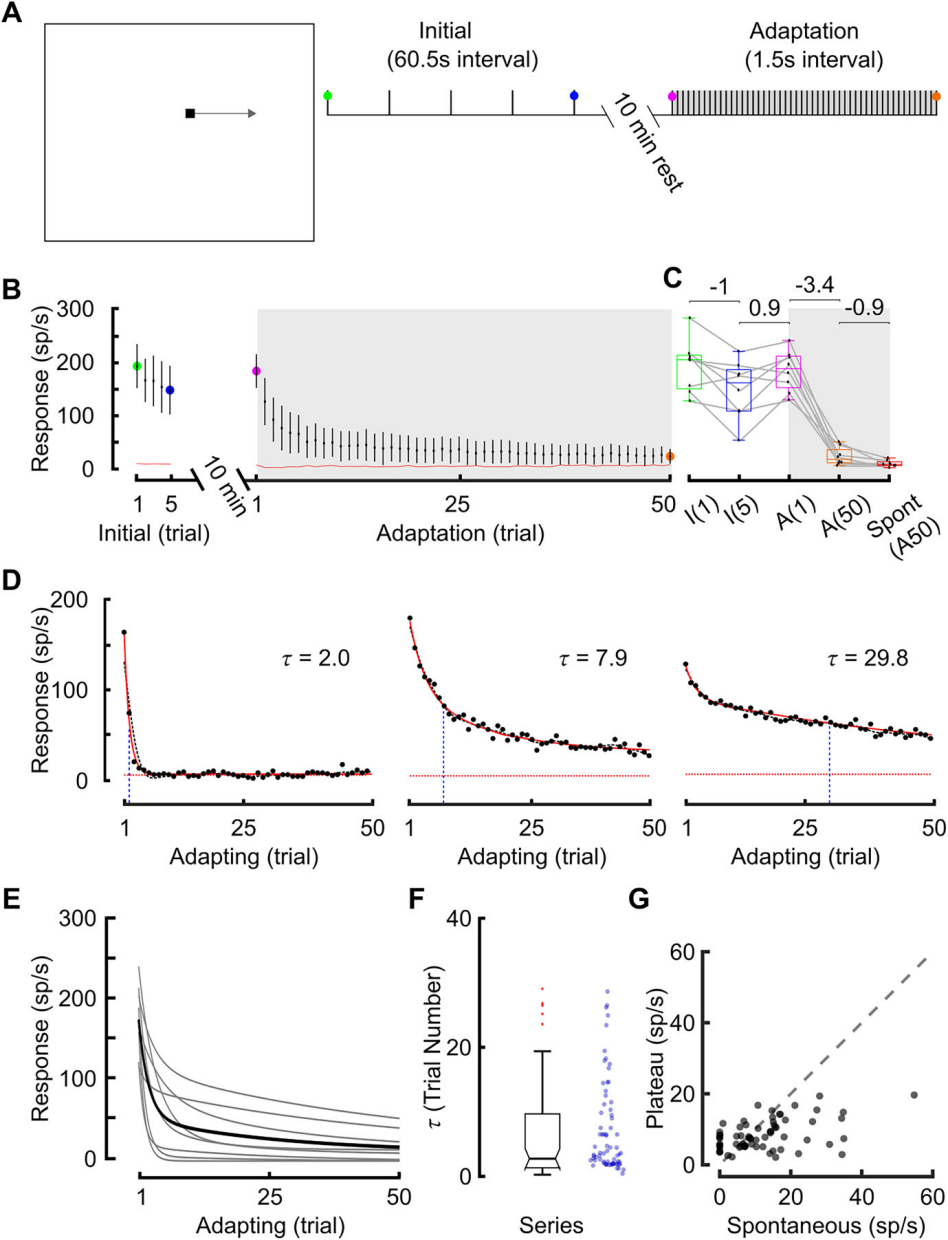
Characterizing the temporal dynamics of STMD adaptation. ***A***, pictogram illustrating stimulus location. Initial trials had an intertrial interval of 60.5 s (5 trials, with each trial lasting 0.5 s), followed by a 10 min rest. The adapting trials had an intertrial interval of 1.5 s (50 trials). Colored circles denote points-of-interest. The gray box indicates the adapting trials. ***B***, STMDs adapt to a repeating target (*N* = 8, mean ± CI) approaching near spontaneous levels (red line). ***C***, Color coded box and whisker plots show STMD responses at points of interest. During the initial period, there is weak adaptation (I1 to I5) which returns to baseline after the 10 min rest (A1). Following 50 rapid repeating trials (1.5 s interval), responses are strongly adapted to low levels (A50) nearer to spontaneous activity (“Spont”). Bias-corrected Cohen's *d* label the effect size. Gray box represents adapted data. ***D***, Three examples highlighting variability in the time course of adaptation in different dragonflies. Each time course has been curve fit [(*a*_1_ * exp(−*x*. / *b*)) + (*a*_2_ * exp(−*x*. / *c*)) + *d*]. Black dotted line is a spline fit, red solid line is the curve fit. Red dotted line is the mean spontaneous over the 50 trials, black dots represent raw data, and blue line represents decay constant ( τ). ***E***, Time courses (represented by curve fit) are shown for each dragonfly (gray lines) highlighting the range of adaptation between animals (black line is the mean of all curve fits). ***F***, ***G***, similar to ***D***, we curve fit a much larger dataset (the adaptive stimulus consisted of 50 dark targets moving rightward every 1.5 s, *N* = 71, *n* = 142). ***F***, Box and whisker plot of the decay constant ( τ) across neurons (blue dots represent raw data). ***G***, A scatterplot of plateau values versus spontaneous firing rates, with a unity line for comparison.

To quantify the time course of STMD adaptation, we developed a stimulus sequence that interleaved longer breaks between stimuli (i.e., 10 min “rest” periods) with repeating target stimuli at a naive location within the receptive field (Fig. 2*A*). We first used STMD responses to a set of five repeated targets at long intervals to establish an initial baseline response level (Fig. 2*A*, initial, one target trajectory every 60.5 s). We then allowed the neuron to rest for 10 min before exposing it to a rapidly repeating set of 50 targets (Fig. 2*A*, adaptation, one target trajectory every 1.5 s). We averaged technical replicates (following rest periods) of this experiment at three different locations to account for the spatial inhomogeneity of the receptive field.

Responses to the first five stimuli (Fig. 2*B*) show a modest reduction in response between the first (green point I1) and fifth (blue point I5) presentation. After the 10 min rest, the first stimulus of the adaptation sequence shows recovery to the initial level (purple point A1) followed by rapid adaptation over the 50 trials to a profoundly reduced level (orange point A50). Averaged across eight neurons, there was an ∼83% reduction during the adapting period (gray box) toward spontaneous activity (red line) by the 50th trial (A50). Figure 2*C* boxplot shows distributions with pairwise comparison of the effect sizes between time points of interest (Fig. 2*B*, colored circles). Adaptation to the rapidly repeating targets is observed across all STMDs. However, STMDs in different animals reveal variation in the adapting time course (Fig. 2*D*, three individual examples). To illustrate this variation, we curve fit individual datasets (i.e., each adaptive series). A model combining two exponential decays fitted the data well. Figure 2*E* illustrates the variable time course of adaptation across individual STMD units. The decay begins rapidly (first exponential) and then becomes more gradual (second). The mean curve fit (black line) shows the decay does not reach a plateau and is therefore likely to decrease further with additional trials. In some cases, there is a strong rapid initial decay which reaches zero within a few trials. To examine the variability in the decay constant ( τ), we compiled a larger dataset (*N* = 71) where STMDs were adapted with either no or minimal prior stimulus. The decay constant ( τ) is broadly distributed, indicative of the variation in STMD adaptation in different units (Fig. 2*F*). The plateau value shows that in many cases, adaptation reduces responses to levels indistinguishable from spontaneous activity (Fig. 2*G*).

**Figure 3.**
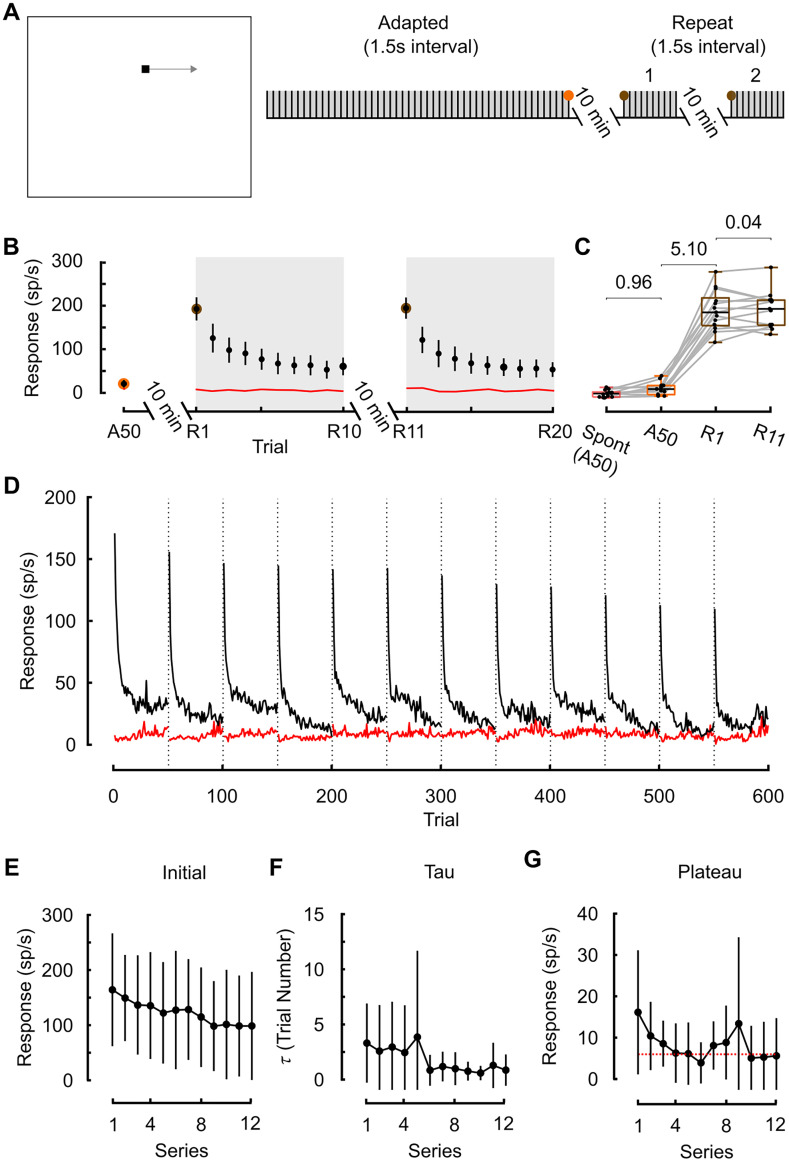
Responses to a repeating adaptation series. ***A***, Pictogram illustrating an adaptive stimulus (50 repeating targets), then 10 repeating targets 10 min later (followed by another 10 repeating targets 10 min following). Colored circles indicate points-of-interest. The gray box indicates the adapting stimuli. ***B***, STMD responses are adapted (orange, A50). After a 10 min recovery period, the mean response (brown points, R1, R11) have recovered to a similar rate as the naive mean response rate (Fig. 2*B*, green circle). The time courses of adaptation look similar during the repeated target series (*N* = 13, mean ± CI). ***C***, Boxplots showing that 10 min after adaptation (A50) responses have recovered (R1). 10 min later again, responses have recovered (R11). Values are bias-corrected Cohen's *d* to show effect size. ***D***, Measuring adaptation over 12 repeats of an adaptive stimulus, each following a 10 min rest indicated by a dotted vertical line. Each of the 12 adaptive series consisted of 50 dark targets, 1.5 s intertrial interval, the black line represents the mean response, and red line represents the mean spontaneous response (*N* = 6). ***E–G***, Data from the repeating adaptive series in ***D*** were curve fit (as per Fig. 2*D*). Linear regression analysis indicates that data in ***E*** and ***G*** is not significantly different from zero at the 95% confidence level (*p* = 0.06 and 0.15), while the slope for ***F*** is, indicating a statistically significant downward trend for the τ (*p* = 0.03).

The decay constant ( τ) had a standard deviation of 7.43. To determine whether this variability was also present in any one individual STMD, we examined another dataset that consisted of several long recordings from individual units (data shown in Fig. 3*D*,*F*). Here, the standard deviation of the decay constant ( τ) in repeated series was much lower, at 1.4 (*N* = 6). The decay constant within individual STMD units remains relatively stable over time, contrasted with the larger variability between different STMD units.

### Repeated series of targets accumulate more rapid adaptation

In studies on habituation, often an accumulative effect is observed upon further repetition of an adaptation series after a break (Thompson, 2009). To test this, we followed the initial 50-trial rapid adaptive stimulus with a 10 min rest period before twice presenting a further 10 trial adaptive series, again with a 10 min rest (Fig. 3*A*). The repeat sequences show similar mean adaptation curves (compare R1 to R10 with R11 to R20), revealing that given the extent of adaptation, our 10 min period between experiments was sufficient to allow responses to recover to the naive state (Fig. 3*B*). On aggregate, the data shows no evidence of an accumulative effect between the two series (compare R1 with R11), with the first trial of both adaptive stimulus similar (Cohen's *d* = 0.04; Fig. 3*C*). However, in a subset of recordings (*N* = 6), we were able to repeat a more extensive sequence of 12 adaptive trials, each using 50 repetitions of the stimulus and with 10 min rest (Fig. 3*D*). This more repetitive sequence (12 repeats of 50 targets compared with 2 repeats of 10 targets) was curve fit, with the initial value, τ, and plateau compared across the repeating series. Linear regression reveals an accumulative decrease of the decay constant (Fig. 3*F*, τ), with the initial target (Fig. 3*E*; *p* = 0.058), and plateau value (Fig. 3*G*; *p* = 0.15) not significantly changed.

### Recovery from adaptation is nonlinear

**Figure 4.**
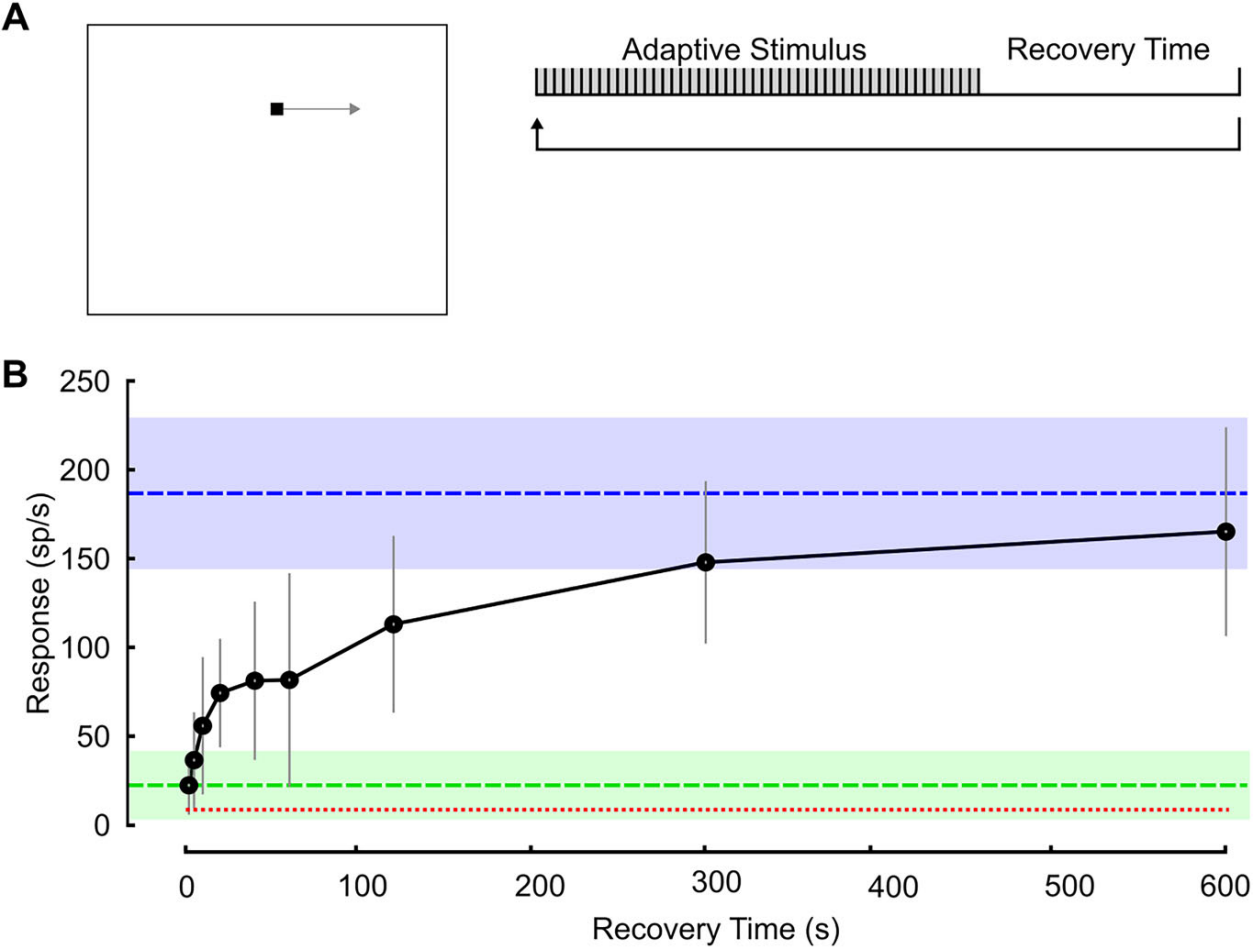
Time course of recovery from adaptation. ***A***, Pictogram showing stimulus location and timing. An adaptive series of 50 repeating targets (every 1.5 s) was followed by a variable recovery time, with the adapting series repeated each cycle. ***B***, Responses for each recovery time plotted on a time axis reveal a nonlinear rate of recovery from the adapted state (mean ± CI, *N* = 7–11 dragonflies). Blue dashed line represents naive responses (mean ± CI). Green dashed line is the level of adapted responses (mean ± CI), and red dotted line is the average spontaneous activity across all trials.

We quantified the time taken for STMD responses to recover from adaptation. After an initial strongly adapting series of 50 targets, we inserted a randomized rest period (2, 5, 10, 20, 40, 60, 120, 300, and 600 s) followed by another adaptive series (Fig. 4*A*). The initial trial from the first adaptive series in each experiment serves as the unadapted response (blue shading). In subsequent adaptive series, following randomized rest periods, the initial trial measures the recovered rate of response. The recovery time is plotted on a time axis (Fig. 4*B*) showing that the recovery from the adapted state is initially rapid, with the end stages of recovery taking longer durations. That is, the stronger the state of adaptation, the steeper the recovery curve, approaching a plateau at ∼10 min (the near-naive state). Recovery times were randomized, therefore minimizing the effect of accumulation induced by the repeating 50 target series as described in Figure 3.

### Adaptation is spatially localized

**Figure 5.**
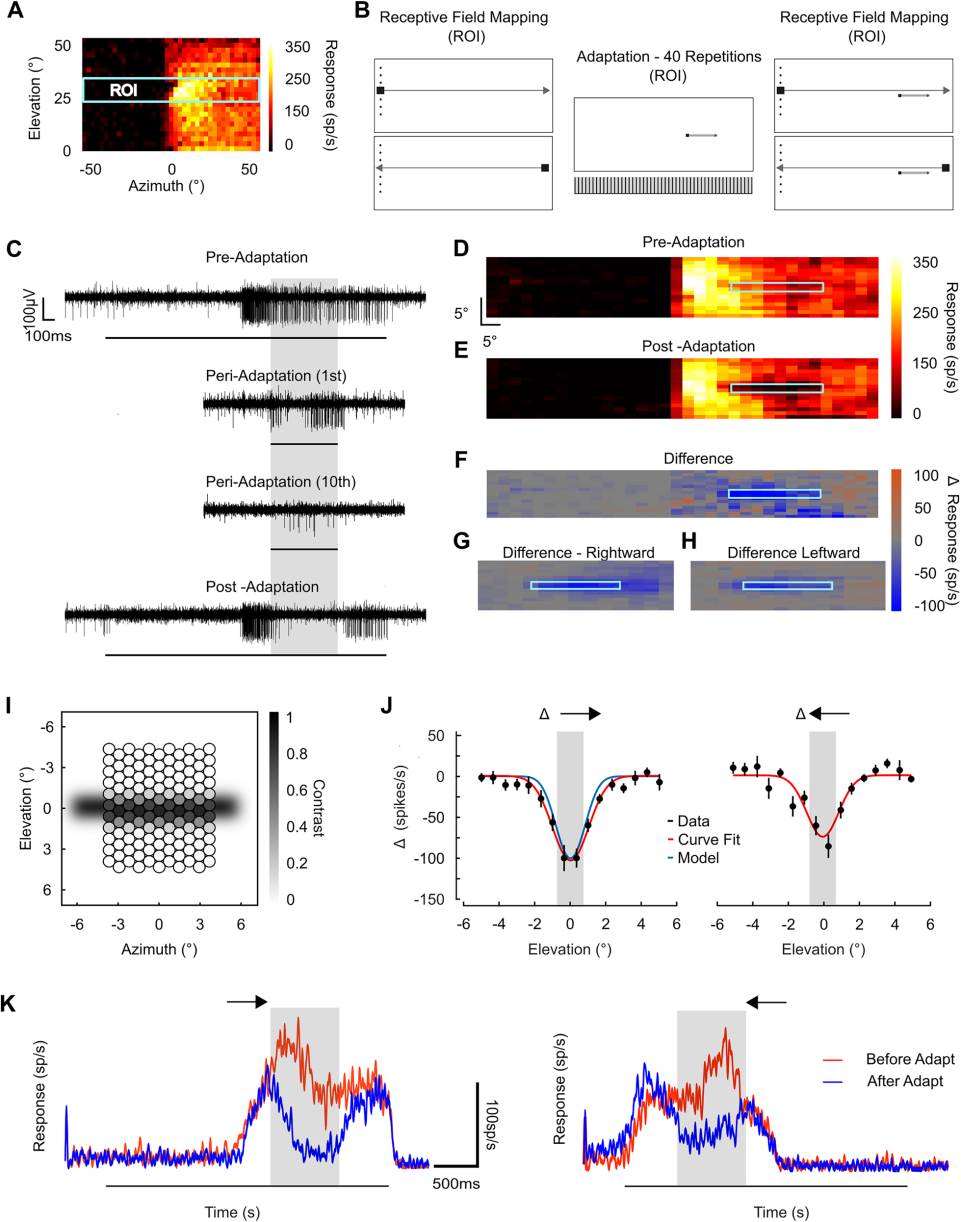
Spatial extent of STMD adaptation is highly localized. ***A***, A region of interest (cyan box) was selected as to maximize rate of response, enabling a higher resolution mapping of the STMD receptive field (104.15° horizontally, and 10° vertically). ***B***, Pictograms illustrating the high-density mapping and adaptation series. Within the ROI, we mapped 32 overlapping and pseudorandomized horizontally moving trajectories (0.9° by 0.9° moving at 30°/s, 0.275° overlap top/bottom, 20 s rest). Within the ROI, we adapted the STMD with 40 dark targets at 1.5 s intertrial intervals (1.5° by 1.5°), moving rightward at 50°/s for 25°/s. A subsequent high-density mapping included adaptation stimuli “top ups” (repeating single targets traversing the adaptive region) during what was the previous 20 s rest periods within the initial receptive field mapping sequence. ***C***, Spike traces (Butterworth filter) of an individual recording showing pre, peri, and postadaptation (gray box represents region of adaptation, black bar indicates peristimulus timing). ***D***, Example of a high-density, rightward mapping of the ROI within the STMD receptive field (32 scans, 100 ms bins scaled to represent equivalent dimensions in space). Dashed lines indicate where the following adapting target trajectory was located. ***E***, Example mapping following the adapting stimuli, where in a localized region spiking activity has been reduced to near spontaneous levels. ***F***, Example of the difference between post and pre-mappings reveals the spatial extent of adaptation. ***G***, Average differences (12 dragonflies) of a rightward mapping (rightward adapting stimuli). ***H***, Leftward mapping (rightward adapting stimuli). ***I***, An optical model illustrating the hexagonal arrangement of the dragonfly ommatidia, its associated spatial resolution and contrast sensitivity, and how these features correlate with the spatial extent of adaptation. The horizontal gray bar underlying ommatidia indicates the optical blur and extent of localized adaptation induced by the passage of repeating target stimuli. The model both demonstrates how different ommatidia perceive varying levels of contrast when a moving object passes by and predicts localization of adaptation by illustrating how the moving target blurs across the ommatidia. It shows that the stimulus predominantly affects a localized set of ommatidia within the full-width at half-max of the spatial extent of adaptation, aligning with observations as to the spatial extent of adaptation. ***J***, Differential color map data from ***G*** and ***H*** is represented here, with only data from the midsection of the adaptive region shown. The gray shaded area indicates the region of adaptation. A Gaussian fit (red line) shows the spatial extent of adaptation induced by a 1.5° × 1.5° target. The blue line (left) shows spatial extent predicted by the model (***I***). ***K***, To observe the response kinetics of the STMD while entering and traversing the adapted region, we averaged the two central probe trajectories (still divided into 100 ms bins), the red line is preadaptation, the blue is postadaptation (both passed through a Butterworth filter—3rd order; cutoff frequency of 0.25), and the gray box shows the adapted region.

Does adaptation represent a global process or is it a local change limited to the specific region where the stimulus is presented? To test this, we probed the spatial extent of adaptation following a local adapting target stimulus (1.5° by 1.5°). We first delineated a region of interest (ROI) within the STMD's receptive field (where the most robust response occurs (Fig. 5*A*, yellow/white region). The receptive field was then carefully scanned in both horizontal directions with vertically offset stimuli at smaller intervals than in the initial characterization (Fig. 1). Within this region, we then adapted a localized trajectory (40 repetitions moving rightward at 1.5 s intervals). We then rescanned the receptive field, while including a similar adaptive stimulus during the mapping rest periods (Fig. 5*B*). This ensured that adaptation was sustained during the mapping process. Mapping the receptive field in both directions allowed us to test if the spatial extent of adaptation as observed with a rightward moving probe was different from the leftward moving probe (i.e., the less preferred direction).

Figure 5*C* shows an individual raw trace both before, during, and after the localized adaptive stimuli. Response reduction within the adapted region is evident by the 10th trial. In the postadaptation case, the neuron responds strongly both before and after traversing this adapted strip (Fig. 5*C*, bottom trace). Figure 5*D* shows an exemplar high-density receptive field preadaptation (the dashed box indicates the location of the future adapting target). Figure 5*E* shows the same individual high-density receptive field map postadaptation, revealing a localized, dark notch of suppressed spiking activity. To further visualize the spatial extent of this adaptation, we plotted the difference between these two maps, revealing the change induced by the adapting series of targets (Fig. 5*F*). The average difference (*N* = 12 dragonflies) is shown for the rightward receptive field (Fig. 5*G*) and leftward receptive field (Fig. 5*H*).

To further describe the spatial extent of adaptation, we first applied a model that accounts for optical blur in the retina and the hexagonal organization of the ommatidia that sample the visual stimuli (D. C. O’Carroll and Wiederman, 2014). This allowed us to predict interaction between the 1.5° adapting stimulus and the 0.9° probe targets. Figure 5*I* shows the ommatidial mosaic predicted by our model, based on ommatidial axis maps of the frontal dorsal acute zone (Horridge 1978; interommatidial angle of 0.7°) and intracellular recordings (D. C. O’Carroll and Wiederman, 2014 and unpublished data; acceptance angle of 1.0°). The blurred gray bar shows the optical blur induced by the passage of an adapting target across the ommatidia. Gray levels within each ommatidium indicate the relative contrast of the stimulus as sampled from the blurred image. Note that due to the orientation of the hexagonal sampling mosaic, alternating columns sample the image differently, while the stimulus extends across several ommatidia in the orientation orthogonal to its trajectory. If we assume that adaptation is linearly proportional to the local strength of stimulation, in that this ommatidial image represents a “memory” or neural afterimage of the passage of the adapter, we can then predict the interaction with the smaller probe feature (the mapping stimulus). We implemented this by multiplying the value of each ommatidium from two adjacent columns of the afterimage with values from a corresponding model for the probe feature moved in small elevation increments across it.

Figure 5*J* (black points and red lines) reveals the spatial extent of adaptation (for both leftward and rightward mapping probes) by averaging the data (Fig. 5*G*,*H*) across a 250 ms window located central to the adaptive region (gray shaded area). The full-width at half-maximum was 2.4° for the rightward and 2.0° for the leftward traveling probes. Thus, the adapted region is very localized, not much more than the size of the adapting target (1.5° × 1.5°). Note the model fit (blue line, left figure) is very close to the curve fit of the data (red line, left figure).

To examine the STMD response kinetics as a small dark target enters, traverses, and leaves the adapted region, we averaged the two central horizontally traversing probes, both moving rightward (Fig. 5*K*, left) and leftward (Fig. 5*K*, right). Nordström et al. (2011) showed that STMDs exhibit a response offset (time constant of 46 ms) when vertically drifting targets cease motion within the receptive field. However, we observe that as the probe enters, the adapted region (gray shaded area) responses decrease slowly toward spontaneous levels over 250 ms. Following the traversal of the adapted region, the response to the target builds slowly over time, similar to previous observations of facilitation following an occlusion over similar space and time (Nordstrom et. al., 2011; Dunbier et. al., 2012; Wiederman et. al., 2017). From this we note that if a response to a moving small target is not maintained, then neither is the neuron's facilitated state.

### Higher frequencies of repeated targets converge to lower sustained responses

**Figure 6.**
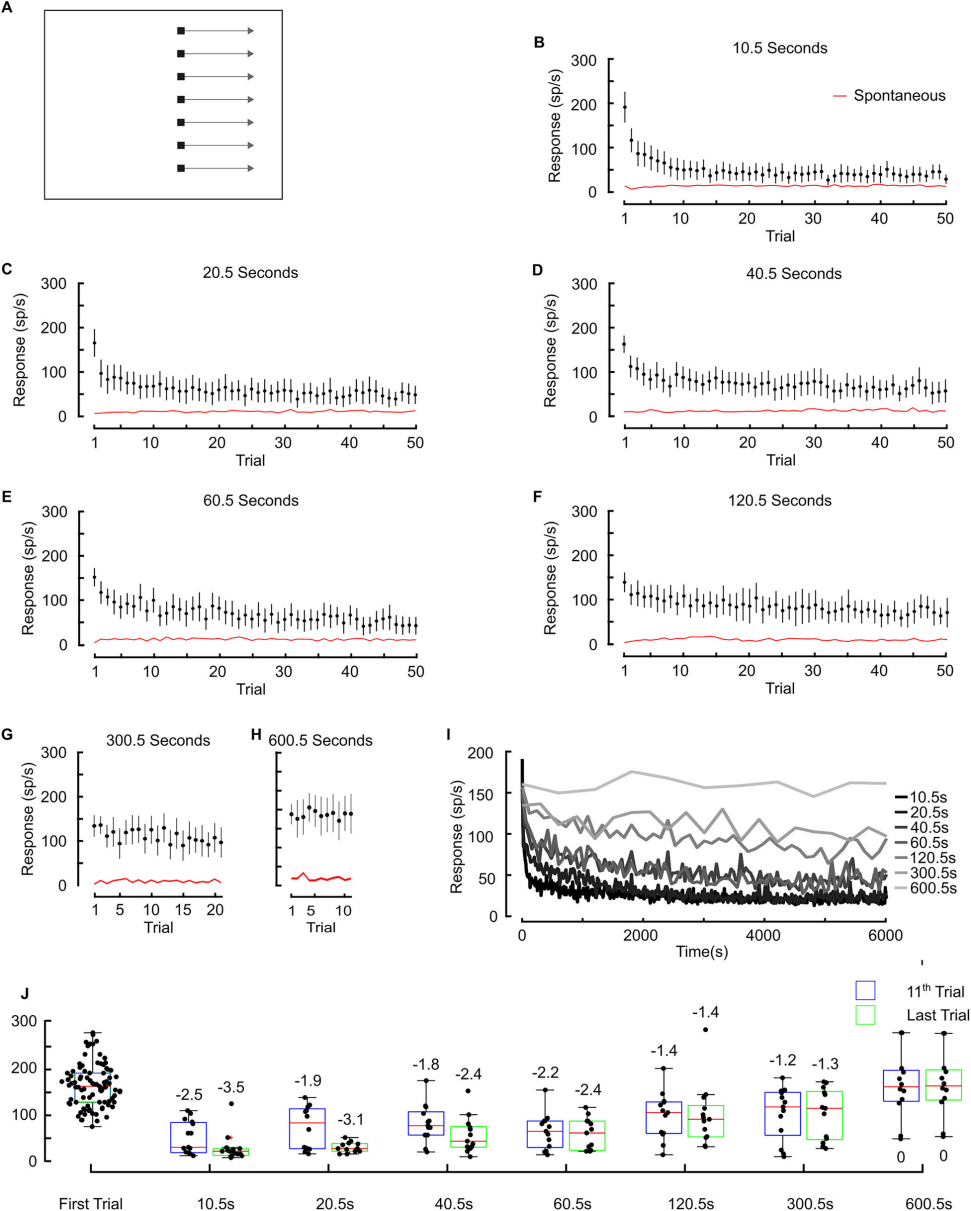
The rate of adaptation is associated with the frequency of the repeating target. ***A***, We presented repeating trials of small moving dark targets on seven trajectories (rightward at 50°/s for 25°). There was only one target on screen at a time, with at least 1 s rest. Each trajectory's intertrial interval was a different duration (10.5, 20.5, 40.5, 60.5, 120.5, 300.5, 600.5 s). The location for each adaptive series with varying intertrial interval was pseudorandomized between animals (trajectories extended 25°; each separated by 4.5°) to account for receptive field inhomogeneity. ***B–H***, The response to each frequency (black lines show mean ± CI; red lines indicate spontaneous activity; *N* = 12–15 dragonflies). ***I***, Mean response rate for each frequency showing the rate of adaptation over time. ***J***, Box and whisker plots show the amount of adaptation between the 11th (blue) and the last trial (green). For intervals of 60.5 s or higher, the STMD reaches a sustained level of response activity (values are bias-corrected Cohen's *d*).

We have revealed that adaptation accumulates over repeated presentations at short (1.5 s) intervals and may take many minutes to fully recover after a long sequence of rapidly presented targets. Is such long-term adaptation driven by the number of repeated trials, or by the interval between them, or both? To address this question, we probed the amount of adaptation induced by varying the frequency of an adapting series. Our experimental design exploited the localized extent of adaptation to present adapting targets in different locations of the receptive field separated by 4.5° (Fig. 6, naive location), with seven different stimulus frequencies.

Figure 6*A* illustrates the seven trajectories used with varying intertrial intervals of adapting stimuli randomly assigned to the locations across dragonflies. Figures 6, *B–H*, shows the rate of adaptation for targets presented every 10.5, 20.5, 40.5, 60.5, 120.5, 300.5, or 600.5 s. For a given spatial location, a 10.5 s intertrial interval induces a rapid rate of adaptation to near-spontaneous levels. For intertrial intervals of 600.5 s, responses to each stimulus can be considered naive. Between these stimuli differing in rate of occurrence, STMD responses reach varying levels of sustained response activity that are then slowly adapting, a nonlinearity similar to that observed in recovery responses. Because this experiment is randomized across vertical locations within an inhomogeneous receptive field, the inclusion of data from less sensitive parts of the receptive field resulted in lower overall activity compared with targets presented in the receptive field “hotspot.” This is why a target repeated at a single location every 20.5 s (conducted at a different spatial location across different animals) gives a mean initial response of ∼160 spikes/s, followed by a slow decay of target responses to a sustained ∼60 spikes/s (Fig. 6*C*).

Figure 6*I* shows aggregate data on a time axis rather than the number of trials, for comparison of time courses of adaptation across frequency of intertrial interval. These time courses reveal a transient decrease in responsiveness until reaching a sustained activity which is no longer adapting. For each variable of intertrial interval, we plotted the 11th and last trial for comparison (Fig. 6*J*) showing that as frequencies increase over 60.5 s, responses converge to a plateau by the 11th trial. Even at very long intervals (300.5 s), we observe a degree of adaptation in the first few trials before neuronal activity reaches sustained levels. Therefore, STMDs may still respond to new targets, even when adapted with previous target motion, dependent on the frequency of their presence.

### Moving targets, rather than luminance or movement, induce STMD adaptation

**Figure 7.**
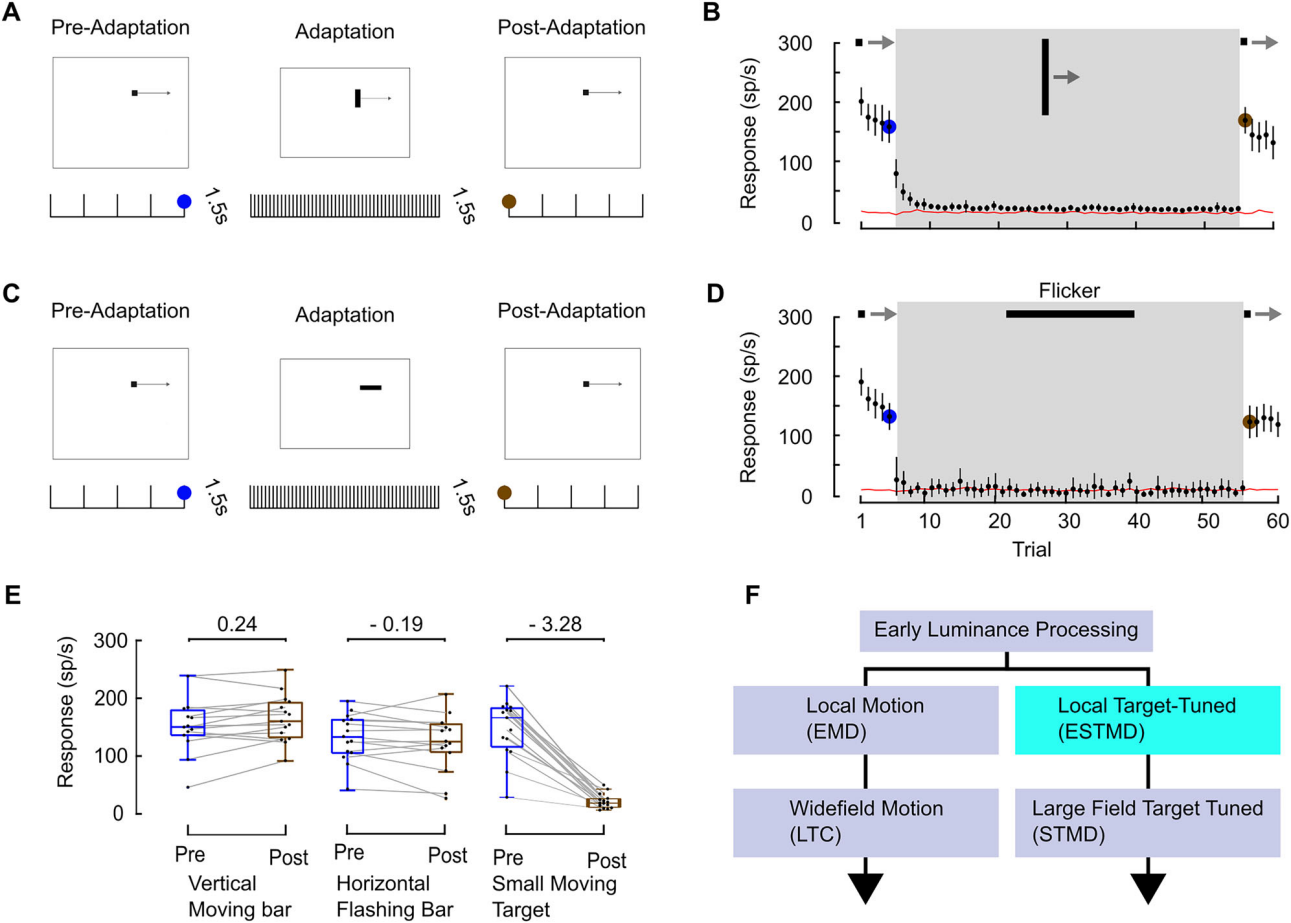
Adaptation is induced by a moving target, not simply by luminance or motion. ***A***, Pictogram, testing if adaptation is caused by a large bar moving in the preferred direction. Five preadaptation trials (dark target moving rightward at 50°/s for 25°), then 50 vertical preferred direction moving bars (25° × 1.5° moving rightward at 50°/s); followed by five postadaptation small target trials. ***B***, Vertical moving bar weakly stimulates the STMD, with responses rapidly adapting. The subsequent response to a small drifting target is not adapted (black lines show mean ± CI; red lines show spontaneous activity; *N* = 15). ***C***, Pictogram, testing whether adaptation is caused by a stationary flickering bar. First five repeats of a dark target moving rightward at 50°/s for 25° followed by 50 stationary flashing horizontal bars (1.5° × 25° flashing every 30 ms, 50 times). Lastly, five repeats of a dark moving target. ***D***, Horizontal flashing bar does not stimulate the STMD and also does not affect the following target response (black lines show mean ± CI; red lines show spontaneous activity; *N* = 15). ***E***, Extent of adaptation between conditions as box and whisker plots reveal that only the rapidly presented target elicits adaptation (the numbers are bias-corrected Cohen's *d*, small moving target data is from Fig. 2*B*, magenta and orange circle). ***F***, Pictogram illustrating a reductive model of the wide-field and feature detection pathways, suggesting a likely candidate for adaptation site (cyan).

Where along the target-detecting pathway is this adaptation likely to occur? In principle, this could occur through local contrast adaptation, even at early stages of visual processing before target selectivity arises. We tested this by examining whether other features might elicit the localized adaptation. We presented a moving bar to determine whether the adapting stimulus must be within the size-tuned range for STMDs (Fig. 7*A*) or if it can still be elicited by a larger feature that exposes the adapted region to equal (or stronger) local motion cues. We also tested a flickering luminance signal composed of a flashing horizontal bar aligned with the tested strip (Fig. 7*C*), a stimulus that would produce strong contrast cues to stages even before any local motion detection.

As expected, the moving bar elicits only weak responses during the adapting period (due to STMD size-tuning). More importantly, although the responses to repeated presentation of the bar do decline further on repeated presentation, this exerts no adaptive effect when an excitatory target stimulus reappears (Fig. 7*B*, compare brown and blue dot). As expected, the STMD shows no response to the flashing stationary horizontal bar. We observe that there is no adaptation induced by flicker on the subsequent target motion (Fig. 7*D*). That is, neither the flashing bar nor bar movement induces STMD adaptation (Fig. 7*E*). We infer that adaptation must follow the neural processing responsible for target selectivity and before any larger-field integration of these units (Fig. 7*F*), likely the hypothetical elementary STMDs (ESTMDs) described in previous modeling efforts (Wiederman et al., 2008).

## Discussion

Investigating STMD adaptation in the dragonfly required long-lasting experiments that permit neuronal responses to return to a naive state. We therefore utilized extracellular techniques, recording from units within the optic lobe that exhibited response properties of STMDs. In this dataset, units had a large, contralateral receptive field, similar to those observed with intracellular recordings from other large field STMD neurons (D. O’Carroll 1993; Geurten et. al., 2007; Evans et. al., 2020). However, these unit responses were not identified to a particular neuron; therefore, variation in adaptation time courses could either represent recordings from different STMD neurons or from the same neuron affected over time by other neuromodulatory factors. For example, rates of adaptation might be modulated via hormonal or behavioral states (e.g., satiation, sleep cycles), though we did not observe strong changes over the time course of our experiments. Across dragonflies, the strength and speed of adaptation could appear both stronger, weaker, faster, or slower than that typically encountered in intracellular recordings. In all cases, responses to a rapidly presented target elicited initial strong responses, decreasing over a small number of trials until reaching a sustained level of reduced responsiveness. How this transiently strong and weaker sustained activity relates functionally to relevant visual scenarios will require further experimentation.

Although adaptation time courses to short interval stimuli were different across animals, they were relatively consistent over a repeating adaptation series. Only the decay constant ( τ) slightly decreased, indicating a small effect akin to habituation or fatigue (Thompson, 2009). We did not observe sensitization or dishabituation typical of other systems (Fraser Rowell, 1971). While most of the recovery from adaptation was initially rapid, later periods required longer durations to recover. That is, the nonlinear recovery times increased at a decreasing rate toward the initial response activity (out to 10 min). Whether there are any functional implications of this long-lasting accumulation is yet to be determined.

Neurons typically adapt; however, the role adaptation might play varies in different pathways. For example, adaptation in locust visual neurons resembles the “oddball paradigm,” where neural activity decreases in response to repeating stimuli until novel stimuli appear, eliciting a robust response (Fraser Rowell, 1971; Weber et al., 2019). In both early vision and motion pathways, adaptation may rescale responses to match their limited dynamic range to fluctuations in the statistical properties of visual inputs (Brenner et al., 2000; Fairhall et al., 2001; Zheng et al., 2009; Weber et al., 2019; Drews et al., 2020). In contrast, STMD adaptation is likely driven by the strength of response to stimuli (i.e., salience), more akin to “spike frequency adaptation” where there is a decay in response over time, not reversible by novel stimuli (Weber et al., 2019).

The reduction in STMD activity in response to repeated, salient stimuli has a time course comparable with other systems, such as the ferret primary visual cortex (Sanchez-Vives et al., 2000), the dragonfly ventral nerve cord (Olberg, 1981), and the locust descending contralateral movement detector (Palka, 1967; Horn and Fraser Rowell, 1968; Fraser Rowell, 1971). A repeating dark target elicits adaptation relative to the frequency of the stimulus, with activity converging to a sustained level. The above rates of adaptation are slow (∼83% reduction after 75 s) in comparison with other descriptions of adaptation in visual sensory neurons. For example, photoreceptors (Weckström, 1989) and motion-sensitive lobula plate tangential cells (LPTCs; Maddess and Laughlin, 1985; Harris et. al., 1999) have adaptation time courses and recovery periods ranging from tens to hundreds of milliseconds. However, photoreceptor and LPTC adaptation is elicited by a continuous stimulus, whereas our target stimuli are discontinuous by necessity. Interestingly, adaptation in photoreceptors and LPTCs make them more responsive, as responses are relieved from saturation, providing a greater response range to signal changes (Maddess and Laughlin, 1985; Harris et. al., 1999). However, in the target pathways, adaptation to repeated targets suppresses spiking activity nearer to spontaneous levels.

In some neuronal pathways, adapted responses may be returned to a naive state by displacing the location of the stimulus (Palka, 1967; Horn and Fraser Rowell, 1968; Fraser Rowell, 1971; Olberg, 1981). Similarly, we have shown in dragonfly STMDs that adaptation is very localized, to the level of the ommatidial mosaic of the compound eye (Horridge, 1978), with a spatial extent confined to a 2.4° half-width. Thus, localized subregions of the STMD receptive field can have differing states of adaptation at any moment, dependent on the previous stimulus statistics.

Such localization is also observed in adaptation of retinal ganglion cells in the larval tiger salamander (Kastner and Baccus, 2013). However, unlike these neurons, we did not observe an increase in sensitivity surrounding the site of local adaptation.

The size of the adapted region corresponds to that of hypothetical ESTMDs, previously used to model the properties of STMD neurons (Wiederman et. al., 2008). ESTMD modeling simulates target selectivity within a retinotopic, columnar structure. In the optic lobe, the medulla is the last level of retinotopic organization where columnar neurons represent a 1:1 projection from the retina (Strausfeld and Campos-Ortega, 1977; Ito et. al., 2014). This suggests that the STMD adapting elements are more likely to be medullary (or earlier), columnar neurons, rather than downstream in the lobula complex where STMDs are located.

STMD responses to adaptive stimuli composed of moving targets, moving vertical bars and a horizontal flashing bar, provided additional evidence for the likely site of STMD adaptation. This adaptation cannot be in the retina, as otherwise the animal would go blind to any stimulus. However, there are multiple lamina and medulla pathways (Strausfeld 1971; Armett-Kibel et al., 1977; Meinertzhagen and Armett-Kibel 1982; Meinertzhagen and O’Neil 1991; Briscoe and Chittka 2001) where flickering stimuli may have played a role in adaptation. Instead, we show that adaptation is driven by target-selective elements at the scale of the ommatidial mosaic, rather than luminance or motion signals (for modeling of the visual pathways, see Wiederman et al., 2008; Nordström and O’Carroll, 2009; for a review of modeling of pathways, see Nordström, 2012).

Pursuits by perching dragonflies are typically quick (<300 ms) with targets fixated in their optical acute zone (Olberg et al., 2000, 2007; Mischiati et al., 2015; Lin and Leonardo 2017). The result of such closed-loop pursuits will be a target that rapidly and repeatedly moves over a local region of the eye, thus potentially eliciting STMD adaptation. *H. tau* are hawkers, patrolling territory and pursuing prey and conspecifics over longer durations than perches. Given the duration and form of these patrols, adaptation is likely to play an even more important functional role. The question arises as to whether a repetitive target motion across the dragonfly eye during pursuits induces apparent blindness to the target? How such long-lasting, naturalistic stimuli that corresponds to dragonfly neuroethology elicits STMD adaptation is a topic for future investigation.

## Synthesis

Reviewing Editor: Michaël Zugaro, CNRS, Collège de France, Inserm Decisions are customarily a result of the Reviewing Editor and the peer reviewers coming together and discussing their recommendations until a consensus is reached. When revisions are invited, a fact-based synthesis statement explaining their decision and outlining what is needed to prepare a revision will be listed below. The following reviewer(s) agreed to reveal their identity: Max Turner. Note: If this manuscript was transferred from JNeurosci and a decision was made to accept the manuscript without peer review, a brief statement to this effect will instead be what is listed below.

All concerns have now been addressed.
